# Supplementary material for: Are carbon emissions trading and green financial instruments synergistic? -Comprehensive quantitative research based on content analysis
Source: PLoS One. 2024 Mar 7;19(3):e0298601. doi: 10.1371/journal.pone.0298601 (PMC10919601; doi:10.1371/journal.pone.0298601)
Supplement: S1 Data — (ZIP) [file pone.0298601.s001.zip › Data/地级市Z_F工作报告.zip!/╣π╬≈/╣≤╕█╩╨/└┤╫╘╣1⁄2╓┌║┼í╛┬φ┐╦╩2╛▌═°í┐_2014.docx]

各位代表：

现在，我代表市人民政府向大会作工作报告，请予审议，并请各位政协委员和列席会议的同志提出意见。

一、2014年工作回顾

过去的一年，在自治区党委、政府和市委的正确领导下，在市人大及其常委会、市政协的监督支持下，我们紧紧依靠和团结全市人民，真抓实干，开拓进取，攻坚克难，全市经济社会实现平稳健康发展。初步核算，全年地区生产总值805.4亿元，增长5.2%；财政收入66.11亿元，增长15.1%，增幅在全区排名第1位；全社会固定资产投资611.4亿元，增长23.2%，增幅在全区排名第1位；外贸进出口总额3.06亿美元，增长38.3%，增幅在全区排名第3位；城镇居民人均可支配收入、农民人均纯收入分别增长8.9%和11.5%，均高于全区平均水平；其他经济指标也保持较好的增长，各项社会事业协调发展。

（一）经济发展质量进一步提升。以“1010”工作为抓手，通过抓重点、创典型、出亮点、争先进，推动经济稳中有进、进中向好。产业结构进一步优化，三次产业结构调整为20：40.4：39.6。财政收入质量继续提升，税收收入占财政总收入的85.2%，非税收入占一般公共预算收入的26.9%。工业经济持续发展。规模以上工业总产值796.8亿元，增长8.1%；增加值246.2亿元，增长5.4%。新增规模以上工业企业29家，总数达404家；新增年产值超亿元企业12家，总数达182家。节能工作成效明显，万元工业增加值能耗下降7.2%，预计万元地区生产总值能耗下降4%。农业稳步发展。农林牧渔业总产值278.7亿元，增长3.1%；粮食产量154.8万吨，实现“十连增”；特色经济作物种植面积319.7万亩，新增6.4万亩；土地流转试点工作稳步推进，实现水田“以小并大”面积6.2万亩；新建“菜篮子”基地13个，实施“双高”糖料蔗示范基地1万亩；新增市级以上农业龙头企业30家，总数达107家；新增农民专业合作社232家，总数达1392家；新增家庭农场175家，总数达866家；新增规模养殖场42个，总数达575个；大力实施农业品牌战略，新增“三品一标”农业品牌46个、富硒农产品生产试点基地20个。农业机械化水平进一步提升。服务业加快发展。社会消费品零售总额359.6亿元，增长11.8%。旅游业健康发展，接待游客人数增长15.5%，旅游总收入增长25%。港口货物吞吐量5242万吨，增长7%，其中集装箱12.3万标箱，增长9.6%。美家居国际博览城、西江华轩物流中心等一批商贸项目和金田起义博物馆、西山泉旅游文化综合区等重大旅游项目开工建设。贵港西江综合保税区申报和规划建设工作稳步推进，获批设立四个保税仓，其中两个已投入运营。金融服务经济发展能力进一步增强。全年辖区金融机构各项存款余额905.75亿元，比年初增长10.8%；各项贷款余额546.92亿元，比年初增长13.9%。融资在保余额26.28亿元，担保在保发生额24.8亿元。保险、邮政、电信等其他服务业实现较大发展。

（二）项目建设取得新成效。狠抓“三年目标任务行动计划”，组织实施了663个重点项目，完成投资239.1亿元，实现竣工139个。其中市级统筹推进的30个重大项目，完成投资34.6亿元，全面完成形象进度。2014年是实施“三年目标任务行动计划”的收官之年。三年来，我们坚持打基础、利长远，统筹推进港口、园区、城建、农业、民生等五大方面项目建设，截止2014年底，共实施项目1352个，累计完成投资629.4亿元，实现新开工项目852个、竣工605个。其中，市级层面统筹推进项目677个，累计完成投资443.6亿元，实现新开工项目314个、竣工304个，全面实现了“一年打基础、两年见成效、三年上台阶”的目标。港口方面，共组织实施了20个项目，实现新开工7个、竣工7个，累计完成投资12.4亿元，贵港航运枢纽二线船闸、武林作业区二期等重大项目开工建设，南宁至贵港二级航道、贵钢专用码头等一批项目建成投入使用，水运基础设施条件得到进一步改善。园区方面，共组织实施了187个项目，实现新开工82个、竣工89个，累计完成投资204.4亿元，建成标准厂房180万平方米，引进了10个年税收超亿元的产业项目，启动建设粤桂（贵港）热电循环经济产业园、钢材加工贸易物流园、有色金属新材料产业园等特色园中园，西江重工一期、隆昌纺织一期等一批项目建成投产，园区经济成为拉动全市经济增长的主引擎。城建方面，共组织实施了280个项目，实现新开工103个、竣工88个，累计完成投资185.6亿元，建成了贵港、桂平、平南三个高铁站和市图书馆、博物馆、民族文化公园等一批重大城市配套设施，市生活垃圾焚烧发电厂点火运行，中心城区“天网工程”二期竣工运行；西南大桥、同济大桥、高铁贵港站前广场、市体育中心、市文化艺术中心、市中心医院等一批重大项目实现开工并顺利推进，城市功能不断完善，城市品位进一步提升。农业方面，共组织实施了103个项目，实现新开工68个、竣工57个，累计完成投资22.4亿元，大藤峡水利枢纽工程实现开工，华晟木业、城乡现代流通网络工程等一批农田水利、农林产品加工和流通项目竣工，农业发展基础进一步夯实，农业产业化不断加快。民生方面，共组织实施了87个项目，实现新开工54个、竣工63个，累计完成投资18.8亿元，新建了市社会福利中心、市精神卫生医院、港南二中等一批民生项目，公共服务能力明显提升。

与此同时，招商引资取得新成绩。全年新引进项目233个，合同投资额244.46亿元；实际利用外资2200万美元，增长8.1%；广东广业、上亿集团等一批知名企业入驻。

（三）城乡面貌呈现新变化。坚持规划引领，完成了中心城区8个片区的控制性详规和7个乡镇、272个村屯的总体规划或控制性详规。加快城市建设步伐，中心城区至覃塘一级路、江南大道等一批项目实现开工，东环郁江大桥、同济大道下穿铁路工程、解放路下穿铁路工程等一批续建项目加快推进，打通了仙衣路、迎宾大道东侧支路等9条城区“断头路”，城区交通状况逐步改善。深入开展市容市貌集中大整治活动，城区面貌焕然一新。严厉打击“双违”行为，共拆除违法建筑面积67万平方米。全市城镇化率达47.3%，比上年提高了2.5个百分点。狠抓“县县通高速、市市通高铁”建设，南广高铁、桂平至来宾高速公路竣工通车，梧州至贵港高速公路（贵港段）已具备通车能力，贵港至合浦、梧州至柳州高速公路（贵港段）开工建设。加强农村基础设施建设。筹资4.6亿元建设农村公路643公里，全市通水泥路（或沥青路）行政村比例达92%；筹资6.5亿元用于水利工程建设，完成病险水库除险加固37座、中小河流治理项目8个，新增或改善灌溉面积11万亩；建成农村饮水工程182个，解决了51.2万农村人口饮水安全问题；建设水库移民新村93个，建成农村户用沼气池2000座。深入推进“美丽贵港·清洁乡村”活动，建成58座乡镇垃圾转运站、7100多个村屯垃圾池，基本实现每个自然屯建有垃圾池的目标；加强水源清洁，建立42个清洁田园示范片（点），建成农村生活污水处理设施35座，完成规模养殖场减排治理387家，水功能区达标率100%，饮用水水质达标率100%；加强大气污染防治，环境空气质量优良率达95.9%。国家森林城市创建工作取得新进展，全市森林覆盖率达46. 3%。

（四）社会事业全面进步。教育事业加快发展。新建和改造校舍42万平方米，市达开高中扩建工程一期、荷城二小、荷城三小、荷城一幼等城区学校实现开工，完成续建、改扩建或增设幼儿园370个。全市学前三年毛入园率76.2%，九年义务教育巩固率95.16%。职业教育加快发展，贵港职业学院并入广西工业职业技术学院，2014年实现招生超5000人；西江职教园项目扎实推进，概念性规划通过评审。特殊教育取得新发展，市特殊教育学校建成实现招生。教育改革有序推进，出台了促进教育事业加快改革发展的系列措施，实行了教师轮岗交流制和校长任期交流制，提高了偏远艰苦地区学校教师待遇。大力支持民办教育发展，安排2300多万元用于扶持民办学校教师购买社会保险和相关专项奖补。卫生计生事业稳步发展。积极推进乡村卫生服务一体化管理，实施国家基本药物制度的村卫生室达1004个；稳步提高新农合保障水平，农民参合率达99.09%；医改工作取得新成效，全面实施新农合支付方式改革，桂平市和平南县7家县级公立医院改革试点扎实推进；加强重大疾病防疫防治工作，国家免疫规划“五苗”报告接种率均达95%以上；稳妥实施“单独两孩”政策，人口自然增长率为8.27‰。科技创新能力不断增强。新增授权专利343件，新增国家高新技术企业2家、广西农业科技园区2家、广西工程技术研究中心1家。文化体育事业繁荣发展。举办了首届“美丽贵港·我爱荷城”荷花展系列活动，贵港荷文化、“和为贵”等文化品牌影响力进一步扩大；建成村级公共服务中心99个、农村体育健身工程16个，《贵港客家山歌》等4个项目入选自治区级非物质文化遗产名录。认真抓好民族团结进步工作以及和谐寺观教堂创建活动，促进民族和谐与宗教和睦。深入开展国防教育和双拥共建工作，切实加强人民防空和国防后备力量建设，民兵预备役部队遂行多样化军事任务的能力进一步提高。支持工会、共青团、妇联等人民团体工作，统计、对台、档案、地震、气象、贸促、地方志、外事侨务等各项工作都取得了新进步。

（五）民生保障能力不断增强。公共财政支出更多地向基层和民生倾斜，民生支出达121.1亿元，占一般公共预算支出的82.5%。10大类45项为民办实事项目全部完成。支持创业带动就业，城镇就业新增1.8万人，城镇登记失业率为1.9%，农村劳动力转移就业新增4.08万人。社保扩面扎实推进，“五险”参保总人数117.1万人次，增长4.4%；城乡居民养老保险参保人数135.2万人，增长3.5%。加大社会救助力度，投入4.9亿元，救助困难群众26.7万人；投入1952.7万元，救助灾民20多万人次；筹集1059万元为111.4万户农村住房购买政策性保险。提高企业退休人员、城乡居民养老待遇，调整优抚对象、困难群体补助标准，企业职工退休人员基本养老金实现“十连增”，城镇居民医保财政补助标准提高到年人均320元，城乡低保补助标准分别提高了18.6%和15.6%。不断完善养老服务体系，开工建设五保村75个，建成农村幸福院85个，对高龄老人发放了补助津贴。综合防灾减灾能力进一步增强，开工建设市县两级救灾物资储备库。加快推进保障性安居工程，开工建设保障性住房8173套，基本建成5056套，完成农村危房改造6300户。加大整村推进扶贫开发力度，5.8万农村贫困人口实现脱贫。加强价格监管，居民消费价格涨幅1.8%。着力改善基层干部待遇，市辖三区机关事业单位职工与市级实现“同城同酬”，提高了桂平市和平南县机关事业单位职工津补贴标准。基层民主建设不断加强，圆满完成新一轮村委会和居委会换届选举工作。积极创新社会治理，集中处置了一批信访积案，妥善调处了一批突出矛盾纠纷。不断完善治安防控体系，强化反恐防暴，开展了社会治安环境大整治专项行动，始终保持对犯罪活动的严打高压态势。进一步完善应急管理体系，强化食品药品、安全生产、质量监管和突发事件应急处置，社会保持和谐稳定。

在加快经济建设和社会发展的同时，我们狠抓政府自身建设，政府执行力和公信力进一步提升。深入开展了党的群众路线教育实践活动，通过边学边改、建章立制和督促落实，大力开展专项整治，解决了一批影响改革发展和群众反映强烈的突出问题，“四风”问题得到有力整治，干部作风得到有效改进。深化行政体制改革，完成了卫生、计生、文化、广播电影电视、新闻出版、民族、宗教等部门机构的精简整合。积极深化行政审批制度改革，共取消、承接、下放和调整行政审批事项61项。加快推进工商登记制度改革，新登记公司制企业和注册资本分别增长90.6%和154.8%。自觉接受人大法律监督和工作监督，主动接受政协民主监督和社会监督，人大代表建议和政协提案办结率100%。加强政府信息公开和政务公开。推进政务服务工作，市政务服务中心政务办理提速73.6%，群众满意率达99.9%。加强“三公”经费预算管理，全市“三公”经费同比下降15.5%。完善政府投资项目审批、大额资金管理、绩效考评等有关制度，行政效能进一步提高。严格执行中央“八项规定”和党风廉政建设各项规定，严肃查处违法违纪行为。

各位代表，在宏观形势错综复杂、经济下行压力加大的情况下，我市能够保持经济社会发展良好态势，成绩来之不易。这些成绩的取得是市委正确领导、科学决策的结果，是市人大、市政协监督和支持的结果，是全市人民团结一致、奋力拼搏的结果。在此，我代表市人民政府向全市人民，向各民主党派、工商联、无党派人士、人民团体，向驻贵中区直单位和驻贵部队、武警官兵，向所有关心支持贵港发展的各界人士、各位朋友，致以崇高的敬意和衷心的感谢！

在看到成绩的同时，我们也清醒地认识到，我市经济社会发展还存在不少困难和问题，工作中仍有许多不足。主要是：受宏观经济环境的影响和自身经济基础薄弱的制约，部分企业生产困难甚至停产或半停产，一些重点项目投资放缓，致使经济发展速度回落，地区生产总值、规模以上工业增加值等一些经济指标增幅没有达到预期的目标；缺乏优质的重大产业项目支撑，产业龙头企业偏少，加强后续财源建设、优化产业结构仍有大量工作要做；环境资源约束压力越来越大，节能减排任务越来越艰巨；民生改善与群众期盼还有较大差距，社会管理还面临不少矛盾和问题；政府深化改革、转变职能有待加强，各层面执行力和服务效能需要进一步提升。对于这些问题，我们将高度重视，认真研究，努力解决。

二、2015年工作安排

2015年，是实施“十二五”规划的最后一年，也是珠江—西江经济带上升为国家战略后全面开发建设的第一年。今年政府工作的总体要求是：全面贯彻落实中央经济工作会议和习近平总书记系列重要讲话精神，按照自治区经济工作会议的部署要求，紧紧围绕打造西江流域核心港口城市的目标，大力实施“西江经济带项目建设三年攻坚计划”，加快推进港产城互动融合发展，坚持稳中求进，注重扩量提质，强化改革创新，扩大开放合作，统筹城乡发展，增进民生福祉，促进经济平稳健康较快发展和社会和谐稳定。

今年全市经济社会发展的主要预期目标是：地区生产总值增长8%，财政收入增长8%，规模以上工业增加值增长10.5%，固定资产投资增长17%，社会消费品零售总额增长12%，城镇居民人均可支配收入增长8.5%，农民人均纯收入增长10%，全面完成自治区下达的各项经济社会发展任务。

围绕实现上述目标，我们着重推进以下五方面工作：

（一）强化主导产业培育，促进产业优化升级。

推动工业提质增效。坚持特色化引领，围绕仓储物流、食品加工、现代制造、休闲养生、循环经济等主导产业，大力发展工业配套产业，培育壮大龙头企业。重点推进与广东广业公司合作建设粤桂（贵港）热电循环经济产业园，年内完成园区规划、新糖厂迁建等建设任务；着力推进钢材加工贸易物流园、有色金属新材料产业园、东盟（贵港）家具城等特色园中园建设。切实抓好金茂生物化工扩建、雄森生物科技园等在建重点项目，力争年内产生效益。大力发展循环经济，重点推进特种新材料基地和住宅产业化项目，延伸冶金电力和建材产业链。坚持园区集约化发展，依法依规对低效用地和闲置土地进行清理，进一步加强园区基础设施建设，增强园区承载能力。

提高农业现代化水平。稳定粮食生产，确保粮食安全，全年粮食总产量保持150万吨以上。鼓励农业适度规模经营，继续推进水田“以小并大”和土地流转工作，新建“菜篮子”工程基地5个，建设“双高”糖料蔗基地2.5万亩。引导工商资本合理投资农业，培育发展新型农业经营主体，年内新增农业龙头企业20家以上，新增农民专业合作社示范社20家以上，新增示范家庭农场20家以上。加快推进瑞安食品冷链物流、扬翔屠宰深加工等项目建设。深入推进农业标准化、信息化和品牌化创建工程，抓好全市11个现代特色农业（核心）示范区和港北区国家农业综合开发现代农业园区、国家现代农业示范区项目建设。加强农业科技创新，继续实施“一村一品、百技进百村”活动，提升水稻、甘蔗等大宗农作物生产机械化水平。

大力发展现代服务业。围绕建设西江流域城市的生产配送中心、生活配套基地和区域性航运枢纽的发展定位，制定物流业发展规划，出台具体的扶持政策，推动商贸旺市。规划建设一批大型商贸市场和城市综合体，重点推进美家居国际博览城、上亿国际汽贸博览城等大型项目，新引进一批现代物流龙头企业。鼓励建设集大众化服务网点于一体的社区综合服务中心，培育一批多功能乡镇商贸中心，年内新增商业网点6000个以上。加快发展电子商务，年内扶持20家企业发展电子商务、组织50个以上名特优产品进驻淘宝网和邮乐网，建设一批村级电子商务示范点。提升旅游产业。狠抓旅游策划规划，年内完成贵港市旅游发展规划和重点景区景点规划。重点抓好桂平西山景区、平天山森林公园和南山公园提档升级，加快推进金田起义博物馆、西山泉旅游文化综合区、北帝山生态旅游度假区和北环生态农业廊道等一批重点景区景点建设。加强旅游品牌建设，大力发展乡村旅游、农家乐，促进旅游与文化、民俗、生态融合发展。创新理念，整合资源，实施多元化开发经营景区景点，推动旅游业发展壮大，力争年内接待游客人数增长10%，旅游总收入增长15%。大力发展金融服务业，努力拓展政府出资控股的融资性担保公司、金融服务公司的业务。加强小额担保贷款及小额贷款保证保险工作，完善多层次融资担保体系建设，力争年内全市金融机构新增贷款50亿元以上。

（二）大力实施“西江经济带项目建设三年攻坚计划”，增强经济发展后劲。

全力推进重点项目建设。坚持经济工作项目化，项目工作责任化、具体化，通过项目建设推进产业发展和民生改善。集中精力抓好初步确定的投资额较大、带动力较强的80个重点项目，年度计划完成投资103.3亿元。重点抓好隆昌纺织二期、理昂生物质发电等38个续建项目，力促贵港新朝阳现代农业、桂东南服装商贸城一期等30个项目实现开工；加快推进华电贵港电厂二期、西江航运交易所等12个项目的前期工作，力争早日开工建设。

加快推进基础设施网络互联互通。深入实施西江经济带基础设施建设大会战，年内新开工苏湾作业区进港公路、郁水作业区浙商码头、武林至上渡沿江公路提级改造等3个项目，抓好大藤峡水利枢纽、贵港航运枢纽二线船闸、东山多用途泊位、苏湾作业区一期、棉宠作业区一期、武林作业区二期等在建项目。继续加快以“县县通高速”为重点的交通基础设施建设，力争年内梧州至贵港高速公路实现通车，积极推进贵港至合浦、梧州至柳州等在建高速公路（贵港段）建设，切实抓好荔浦至玉林高速公路（贵港段）和柳州至肇庆铁路（贵港段）的前期工作，争取年内开工建设贵港至隆安高速公路。

（三）深入推进改革开放，释放发展活力和动力。

深化经济体制和投融资体制改革。坚持市场在资源配置中的决定性作用，积极采用政府与社会资本合作(PPP)方式，鼓励民间资本参与公用事业、基础设施等领域建设。深化国有企业改革，推进党政机关与所属企业脱钩，优化市建投公司、宏港兴业集团公司、兴港投资公司管理体制，探索组建市产业园区投资公司、交通投资公司、文化旅游投资公司、农业开发投资公司。深化供销合作社综合改革，提升服务“三农”能力和水平。完善农村融资担保服务体系，探索推广农村“三权”抵押贷款，引导融资性担保公司加大“三农”服务力度。进一步降低市场准入门槛，探索负面清单管理模式，扩大注册资本“零首付”及“延期缴付”适用范围，积极促进民营企业创业创新。

加强财税管理改革。全面贯彻落实新的《预算法》，推行全口径预算和财政预算绩效目标管理，建立完善政府性债务管理及风险预警机制。实行中期财政规划管理，建立跨年度预算平衡机制。深化公务卡制度改革，年内实现市直预算单位公务卡全覆盖。完善市直财政国库集中支付动态监控具体操作管理办法，年内实现乡镇国库集中支付改革全覆盖。完善政府购买服务制度体系，拓展政府购买服务领域和内容。

深化行政体制改革。强化政府公共服务，优化职能设置，规范权力运行。深化行政执法体制改革，进一步推进综合执法。整合基层政务资源，抓好乡镇国土、住建、环保、安监等机构“四所合一”试点。扎实推进撤乡改镇、撤镇改街道办和平南撤县改市工作。稳步推进事业单位分类改革。深化园区管理体制改革，实行公司化管理、企业化运作。组建不动产统一登记工作机构，实施不动产统一登记。

统筹推进其他重点领域改革。继续深化教育领域综合改革，促进教育事业均衡发展。加快推进县级公立医院和新农合保障制度、医保支付方式改革，引导医院控制成本，遏制过度医疗；探索新农合基金市级统筹，建立基金责任分担机制，提高基金统筹能力。稳妥推进机关事业单位工作人员养老保险制度改革。建立市本级与县市基本养老保险基金缺口分担机制，确保基本养老金按时足额发放。按照自治区的部署和要求，扎实推进公务用车改革。深化农村土地改革，加快推进农村土地确权登记工作，规范农村土地经营权流转市场交易。推进政策性农业保险体系建设，加大政策性农业保险覆盖面。

深入推进开放合作。以实施珠江—西江经济带发展规划为契机，深化与周边城市和粤港澳台的区域合作，促进优势互补。加强与深圳市食品工业协会的沟通对接，力争合作共建深圳市农产品贵港种植基地和加工物流园。积极与玉林市合作共建东津物流园区和共同申报西江综合保税区。加大保税仓建设力度，促进加工贸易业发展。组织企业实施“走出去”策略，提高对外贸易水平，力争年内外贸进出口总额增长14%。加快完善口岸大通道体系，启动电子口岸建设，提高通关效率。坚持重力招商、招大引强和产业链招商并重，深入开展“民企入贵”“央企入贵”等招商活动，力争年内新引进投资超亿元项目10个以上，签约项目合同投资总额60亿元以上，实际利用外资增长10%以上。

（四）加强生态文明建设，打造美丽贵港。

统筹城乡发展。围绕港产城融合发展，进一步优化城乡布局规划，推动经济社会发展总体规划、产业发展规划、城市规划和土地利用规划“四规合一”。加快推进同济大桥、西南大桥、中心城区至覃塘一级路、高铁贵港站前广场、堤路园、园博园等重大项目建设，年内东环路实现通车；积极推进以棚户区改造为重点的旧城改造，不断改善老城区居住环境；加大市容市貌集中整治力度，持续打击“双违”行为，提升城市品位。整合现有信息基础设施资源，推动新兴技术与政务管理、市政管理、社会管理紧密结合，加快推进智慧城市建设。加强通信服务设施建设，年内中心城区广场、公园、客运站等公共场所实现免费WIFI上网服务，全市行政村小学互联网覆盖率达100%。统筹城乡基础设施建设，实施病险水库除险加固30座，完成中小河流治理项目8个，新增恢复或改善灌溉面积8万亩；建设农村饮水工程190项，解决45万农村人口饮水安全问题；建设水库移民新村85个，新建农村户用沼气池1850座。

抓好节能降耗工作。坚决淘汰落后产能，健全重点行业和企业的节能减排管理制度，落实能源消费总量和强度“双管控”措施，力争年内万元工业增加值能耗下降4%，万元地区生产总值能耗控制在自治区下达指标内，全面完成“十二五”节能减排任务。

加强环境保护。坚持源头治理、过程严管、后果严惩，加大对生态环境保护考核的力度。认真执行环境评价和“三同时”制度，严把项目建设准入关，对已批准但达不到国家环保标准的项目坚决予以取缔。加强环保基础设施建设，重点推进西江污水处理厂、城区第二污水处理厂、桂平和平南污水处理厂等6个在建项目，年内建成大圩镇、木乐镇等8个乡镇污水处理厂，完成中心城区40公里的污水管网和7个排污口截污工程。继续推进畜禽养殖生态化发展，年内完成150个规模养殖场污染减排设施建设。大力推进机动车污染减排，继续加强PM2.5空气环境质量监测和全流域水质监测，提升预警预报能力。推进国家森林城市创建工作，强化天然林、珠防林保护和建设，重点优化调整林业种植结构，大力发展水源林、混交林，严禁在基本农田、饮用水源保护区、湿地区域种植速丰桉，保护生态环境。

强化国土资源管理。坚持最严格的耕地保护制度和节约集约用地制度，加快永久基本农田划定，保护耕地资源。加强土地矿产卫片执法监督检查，严肃查处违规用地行为。强化矿山企业“三率”考核，推进绿色矿山建设，推动资源开发与环境保护可持续发展。

推进生态乡村建设。继续深入推进“清洁乡村”工作，年内完成69个农村生活垃圾综合示范项目建设、30个田园生态典型示范点建设。全面开展“美丽贵港·生态乡村”活动，年内建成市级生态示范村屯25个，每个县市区各打造100个生态示范村屯。突出抓好村屯绿化，年内建成自治区级绿化示范村屯280个，全市村屯绿化达标30%以上；抓好饮水净化，完成乡镇集中式饮用水源保护区划定工作，建成10个饮水净化示范工程，全市农村自来水普及率70%以上；抓好村屯道路硬化，年内硬化村屯道路600公里以上，基本实现行政村通水泥路（沥青路）目标。

（五）坚持以保障和改善民生为根本，致力增进人民福祉。

切实增加城乡居民收入。继续深入实施“城乡居民收入倍增计划”，推动城乡居民持续增收。统筹城乡就业，建立农民工返乡创业服务和管理体系，探索大学生团队在农村创业就业新模式，鼓励支持自主创业、自谋职业和灵活就业，力争年内城镇就业新增1.5万人，城镇登记失业率控制在4.5%以内。完善农民工工资清欠长效机制，加强劳动监察和争议调解仲裁，构建和谐劳动关系。强化创业培训工作，提升农村劳动力技能，年内农村劳动力转移就业新增3.8万人。创新扶贫开发工作方式，实施精准扶贫，力争年内3.8万农村贫困人口脱贫。

着力提升社会保障水平。加强基本医保、大病保险、医疗救助补充保险的衔接，稳妥推进城乡居民基本医疗保险市级统筹。加强社会保险扩面征缴工作，确保年内“五险”参保人数109万人次以上，城乡居民养老保险参保人员续保率80%以上。加强养老服务，鼓励社会资本投入养老事业。对80周岁以上高龄老人发放补助津贴，力争年内开工建设市老年人活动中心。进一步完善社会救助体系，突出抓好城乡低保、五保供养、医疗救助等专项社会救助工作。加快推进市社会福利中心建设，年内建成75个农村五保村、100个农村幸福院。积极发展慈善事业。加强殡葬管理，逐步免除基本殡葬服务费。规划建设市公益性公墓。扩大农村住房政策性保险覆盖面，提高农村住户抵抗灾害风险能力。加快推进保障性住房建设，年内新建保障性住房7324套，棚户区改造住房9119套，基本建成6000套，分配入住6000套；新建一批农村中小学教师、乡镇卫生院职工和乡镇干部职工的周转房；加快动迁安置房建设，缩短安置周期。

坚持教育优先发展。加快缓解适龄学生“上学难”和“大班额”问题，年内中心城区新开工建设两所小学、两所初中、一所高中，建成荷城二小和市达开高中一期扩建项目，加强桂平市和平南县城区中小学校规划建设。大力发展学前教育，年内新建幼儿园5所，全面完成72所乡镇公办中心幼儿园的建设任务，确保学前三年毛入园率76%以上。积极推进义务教育均衡发展工程，确保九年义务教育巩固率93%以上。大力发展职业教育，继续从资金、土地、师资等方面扶持民办学校，统筹推进其他各类学校建设和发展。

大力发展文化体育事业。建立健全公共文化服务体系，继续推进市文化艺术中心和市体育中心建设，年内市档案馆建成投入使用，新建村级公共服务中心80个。广泛开展群众性文化活动，培育和践行社会主义核心价值观。深入挖掘特色文化资源，支持文艺精品创作，打造以“荷城”为核心的城市品牌。加强文化遗产和非物质文化遗产的保护开发，传承和发展传统优秀文化。加强农村体育设施建设，推动群众体育和竞技体育协调发展。

统筹推进其他各项社会事业。推动科技创新能力持续提升，实施全民发明创造活动，积极推进广西农业科技园区项目建设，新建自治区级以上科技创新平台5个以上。积极发展卫生事业。加快医疗卫生基础设施建设，年内开工建设市儿童医院，完成市中心医院主体工程，建成市全科医生培养基地；大力推行新农合“一卡通”建设，提高补助标准和核销比例，年内新农合参保率保持95%以上。健全诚信计生长效机制，继续加强出生人口性别比综合整治，抓好优生优育，稳定低生育水平，提高人口素质。深入推进民族团结进步创建活动，依法管理宗教事务。认真做好双拥、优抚安置和人防工作，加强国防动员和国防后备力量建设。充分发挥工会、共青团、妇联等群团组织的作用，统筹推进统计、物价、对台、地震、气象、地方志、外事侨务、质量监督等工作。围绕重大工程、重大项目、重大政策组织编制好“十三五”规划。

加强和创新社会治理。全面推进依法治市，增强全社会尊法学法守法用法意识。维护司法公正，做好“六五”普法和法律援助工作。推行社区网格化服务管理，提升群众自我管理和服务能力。完善和创新流动人口和特殊人群管理服务。全面推行重大决策社会稳定风险评估制度，健全社会矛盾纠纷预防化解机制，最大限度地把矛盾纠纷化解在基层、解决在萌芽状态。加强信访网络信息平台建设，着力解决群众合法合理诉求。深入开展“扫黄打非”工作，加大依法管理网络力度。加强食品药品监管，加快市食品药品检验所和食品药品监管系统信息化建设。进一步健全社会监测预警体系，提高防灾减灾和应对突发事件能力。严格落实安全生产“党政同责、一岗双责、齐抓共管”制度，建设全市安全生产监管信息平台，深入开展安全事故隐患大排查、大整治，预防和遏制重特大事故发生。深入推进平安贵港建设，进一步健全立体化治安防控体系，完成中心城区“天网工程”三期和全市乡镇高清视频监控系统建设，严密防范和严厉打击违法犯罪活动，强化反恐维稳工作，不断提升人民群众的安全感和满意度。

三、加强政府自身建设

新形势、新常态对政府工作提出了新要求，我们将坚持为民、务实、清廉，着力打造法治政府和服务型政府。

（一）加强机关党的建设。按照全面从严治党的要求，政府系统各级各部门要坚持党建工作与业务工作同研究、同部署、同落实。认真落实党组织建设工作责任制，加强政府系统机关党组织班子建设，严肃党内政治生活，规范党内组织活动，努力建设坚强的领导集体。加强政府系统机关党员干部党性教育和宗旨教育，引导党员干部牢固树立全心全意为人民服务的意识。加强党的纪律建设，政府系统各级党组织和党员干部都要牢固树立纪律和规矩意识，遵循组织程序，服从组织决定，按制度办事，维护政治纪律、组织纪律和法规制度的严肃性和权威性。

（二）加快转变政府职能。大力简政放权，推动政府职能向公共服务、市场监管、社会管理、环境保护等方面转变。认真制定权力清单、责任清单和负面清单，强化事中和事后的监管。引导和加强社会组织建设，加大政府职能向社会组织转移力度，更好地发挥社会组织协同参与社会管理和服务的作用。加快政务服务平台建设，将市县两级行政审批和服务事项全部纳入本级政务服务中心办理，积极推进并联审批、网上审批，提升审批服务效能，实现承诺办结时限整体提速30%以上。严格落实首问负责制、限时办结制、服务承诺制、责任追究制，坚决整治行政不作为、慢作为、乱作为，为各类市场主体创造公平的发展环境，为人民群众提供良好的公共服务。

（三）全面推进依法行政。实施法治教育、权力规范、依法决策、阳光政务、法治评价等法治政府“五大工程”建设，进一步落实好领导干部学法制度，运用法治思维和法治方式深化改革、推动发展、化解矛盾、维护稳定。强化科学民主依法决策，健全决策机制和决策问责制度。推进权力运行公开化、规范化，完善政务公开、政府信息公开和各领域办事公开制度，促进阳光行政。完善行政执法程序，健全执法责任制和过错责任追究制，严格规范执法行为。自觉接受人大及其常委会的法律监督、工作监督和政协的民主监督，重大决策前及时向人大报告、与政协协商，认真办好人大代表建议和政协提案。强化审计监督，重视公众监督和舆论监督。

（四）努力建设清廉政府。严格执行中央“八项规定”，坚决戒除“四风”，讲实话、干实事，言必信、行必果，着力解决群众最关心最现实的利益问题。认真执行厉行节约反对浪费和公务接待管理的各项规定，严格控制一般性支出，实现“三公”经费零增长。严格落实党风廉政建设责任制，强化惩治和预防腐败体系建设，加强对重要部门、重要岗位和关键环节的权力运行监管，严肃查处违法违纪行为，营造风清气正、廉洁高效的政务环境。

各位代表，新形势、新任务对我们提出了新的更高要求。让我们在市委的坚强领导下，在市人大及其常委会、市政协的监督支持下，紧紧依靠和团结全市人民，同心同德，真抓实干，开拓创新，为把贵港建设得更加美好而努力奋斗！
